# Use of videos to measure dynamic body acceleration as a proxy for metabolic costs in coral reef damselfish (*Chromis viridis*)

**DOI:** 10.1242/jeb.249717

**Published:** 2025-04-10

**Authors:** Kota Ishikawa, Heng Wu, Satoshi Mitarai, Amatzia Genin

**Affiliations:** ^1^ Marine Biophysics Unit, Okinawa Institute of Science and Technology Graduate University, Onna, Okinawa 904-0495, Japan; ^2^ The Interuniversity Institute for Marine Sciences in Eilat and Department of Ecology, Evolution and Behavior, The Hebrew University of Jerusalem, Eilat 88103, Israel

**Keywords:** Oxygen consumption, Fish swimming, Locomotion, Feeding, Respirometry

## Abstract

Quantifying the energy costs of various activities is critical to understand key aspects of animal behavior and ecology. Currently, calorimetry is the most widely used method to measure those costs in laboratory studies, whereas field studies use the doubly labeled water method, heart rate and dynamic body acceleration (DBA). However, these methods are limited or even biased because of restricted space for movement, low temporal resolution and/or the need for logger attachment or implantation. Measuring energy costs of behaviors is difficult, especially in small, highly mobile animals. Here, using a damselfish, *Chromis viridis*, we demonstrate that DBA, obtained from marker-less, automatic video tracking and 3D reconstruction, can effectively estimate oxygen consumption rate. We show that our video-based DBA method can be used to estimate metabolic costs of various activities, such as locomotion and feeding, on an individual basis.

## Teaser

**Summary:** Dynamic body acceleration measured via image processing is significantly related to the oxygen consumption rate of damselfish.

## INTRODUCTION

Animals acquire energy by feeding and allocate it partly to metabolism and activity-related costs. The remaining energy, referred to as net energy gain, is used for growth and reproduction. Thus, in ecology and evolution, it is important to understand energy consumption during specific behaviours. Various methods have been developed to quantify energy costs of animals. Common laboratory methods include direct calorimetry, which measures heat production (Kenny et al., 2017; Lusk, 1932) and indirect calorimetry, which estimates energy expenditure by measuring oxygen consumption (Frappell et al., 1989; Mtaweh et al., 2018). Standard field methods include the doubly labelled water (DLW) method, which measures CO_2_ production using different elimination rates of stable isotopes, or the heart rate method, which uses heart rate as a proxy for energy cost based on calibration curves made in laboratories (Butler et al., 2004; Speakman and Racey, 1988). Despite the utility of these methods, each has its limitations. Laboratory methods are constrained by the size of respirometry chambers. The DLW method is limited in temporal resolution, and the heart rate method is invasive, because it necessitates an implanted logger.

To overcome these limitations, Wilson et al. (2006) developed an effective method that uses dynamic body acceleration (DBA) as a proxy of energy cost. DBA is defined as acceleration associated with animal movements and is obtained by subtracting gravitational acceleration from total acceleration measured with a tri-axial accelerometer attached to an animal (Wilson et al., 2006). The basis of the DBA method is the fact that much of energy consumption by animals is related to movement (Gleiss et al., 2011; Halsey et al., 2009). DBA is strongly correlated with oxygen consumption during activity in a wide range of animal taxa, covering all five classes of vertebrates and some invertebrates (Halsey et al., 2009, 2011; Lyons et al., 2013; Payne et al., 2011; Robson et al., 2012; Wilson et al., 2020). Because of its technical simplicity and generality, this method has been applied in multiple fields, leading to important biological findings, such as the optimized foraging strategy of pumas (Williams et al., 2014), flight cost-dependent survival of juvenile birds (Rotics et al., 2016) and the energy cost-related breeding performance of penguins (Grémillet et al., 2018). However, because DBA measurements require attachment of a logger with an accelerometer and a battery, animals need to be large enough to ensure that the attached instrument does not alter their behaviour (Metcalfe et al., 2016). Many studies have used loggers weighing ∼2–4% of the animal's body weight (Halsey et al., 2009; Wilson et al., 2020). Given that loggers used for the DBA method are often 10–20 g (Brownscombe et al., 2018; Gleiss et al., 2010; Lear et al., 2016; Metcalfe et al., 2016; Noda et al., 2016; Robson et al., 2012), body weight of animals should be at least several hundred grammes. Among vertebrates, animals weighing <100 g represent 51.5% of all mammals, 68.6% of birds, 77.7% of reptiles, 94.6% of amphibians and 52.7% of fish (O'Gorman and Hone, 2012). Therefore, these smaller species should not be overlooked. Furthermore, even when a logger is much smaller than the test animal, it may cause artefacts, especially those due to increased drag during flying or swimming (Pennycuick et al., 2012; Saraux et al., 2011; Vandenabeele et al., 2015; Watson and Granger, 1998).

Here, we propose a video-based method to estimate metabolic costs by measuring 3D DBA via fish body tracking. The method is corroborated by examining the relationship between DBA and oxygen consumption rates. Our results suggest that the proposed video-based DBA method is generally applicable for metabolic studies of small animals.

## MATERIALS AND METHODS

### Fish studied

Small planktivorous damselfish, *Chromis viridis* Cuvier 1830, were used in this study. Fish were purchased from a commercial pet shop (Aqua Planning Co. Ltd, Okinawa, Japan). Five *C. viridis*, designated A to E, with body weights of 6.59, 6.58, 5.17, 8.87 and 9.32 g, respectively, were used in our experiments. Except during trials, fish were kept in a holding tank and fed *ad libitum* with brine shrimp (*Artemia salina*) nauplii. All experiments complied with rules of the Animal Care and Use Committee at Okinawa Institute of Science and Technology Graduate University.

### Oxygen consumption measurements

Oxygen consumption experiments were conducted in a 10 litre recirculating swimming respirometer (Loligo^®^ Systems) with a test section 38 cm long, 10 cm wide and 10 cm deep. A flow straightener was placed at the inlet of the test section to eliminate secondary flow. Flow in the respirometer was driven by an impeller. The frequency of the impeller and streamwise mean flow speeds at the centre of the test section (averaged over an area of 0.93×0.93 cm) were calibrated in the range of 0 to 49.6 cm s^−1^ using particle image velocimetry. The entire respirometer was immersed in a water bath to maintain the water temperature at 25.5±0.5°C. A light (Mitras lightbar 60, GHL, Germany) was placed above the centre of the test section to provide illumination between 07:00 h and 19:00 h (12 h:12 h, light:dark). Experiments were conducted between 27 February and 26 July 2023.

To acquire a range of DBA values, effects of six flow speeds were examined on the five *C. viridis*. Two replicates were performed for each individual at each flow speed.

Fish were transferred individually into the respirometer the night before measurements started, where they were kept at a mean flow speed of 5 cm s^−1^ for 13–15 h. After this acclimation period, we confirmed that the oxygen concentration was stable and that the fish showed normal swimming behaviour, before starting a trial. Each trial comprised 12 measurement cycles at increasing flow speeds from 5 to 30 cm s^−1^ in increments of 5 cm s^−1^ and two measurements at each flow speed. Each measurement cycle consisted of 5 min of flush, 2 min of equilibration and 20 min of measurement following intermittent flow respirometer protocols (Steffensen, 1989; Steffensen et al., 1984). After each trial, fish were removed from the respirometer and were weighed. Oxygen concentration was measured using a dipping probe oxygen mini sensor (Loligo^®^ Systems), and temperature was measured with the temperature probe for witrox (Loligo^®^ Systems) with WitroxView software (Loligo^®^ Systems). To inhibit bacterial growth, the whole tank was treated with bleach solution a day before each trial. Background respiration, measured before and after each trial without fish, was averaged, and this value was subtracted from total respiration rate to obtain the respiration rate from the fish.

Based on the last 15 min of measurement for each cycle, linear regression was conducted between oxygen concentration (mg O_2_ l^−1^) and time (h). Using the slope of the linear regression, *S* (mg O_2_ l^−1^ h^−1^), oxygen consumption rate, *Ṁ*_O_2__ (mg O_2_ kg^−1^ h^−1^) was computed as:
(1)

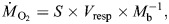
where *V*_resp_ is the volume of the respirometer, and *M*_b_ is the mass of the fish. In this study, *Ṁ*_O_2__ was defined as the oxygen consumption rate normalized by fish mass. Fish behaviour during each 15 min was recorded with cameras to obtain DBA, as described below.

Oxygen consumption rates of the five fish were plotted against the mean flow speed, *U*, and fitted with a three-parameter power function using the nonlinear least squares method (*Ṁ*_O_2__=*a*+*bU^c^*) (Roche et al., 2013). From the nonlinear relationship, standard metabolic rate (SMR) which is the minimum amount of energy required for maintenance or resting metabolic rate was estimated by extrapolation (*Ṁ*_O_2__ at *U*=0; Roche et al., 2013). SMR derived from extrapolation is robust to variations in some fast-swimming fishes (Bushnell et al., 1994; Korsmeyer and Dewar, 2001; Reidy et al., 2000) and is comparable to those measured using a resting respirometer in a coral reef fish species (Roche et al., 2013). The power function and SMR for each individual were also estimated and used to derive the net cost of swimming, defined as *Ṁ*_O_2__–SMR to test its relationship with DBA.

### 3D reconstruction of fish posture

Fish movements were recorded using cameras (acA2000, 165uc, Basler ace, Basler) at 90 frames s^−1^ with a resolution of 1920×1080 pixels to reconstruct body postures in 3D using Direct Linear Transformation (DLT). Cameras were manipulated using PylonRecorder software (https://gitlab.mpcdf.mpg.de/mpibr/scic/pylonrecorder/PylonRecorder), which enables simultaneous triggering of multiple cameras. Two cameras with 25 mm lenses (25 mm C Series Fixed Focal Length Lens, Edmund Optics) were positioned above and on the side of the respirometer.

Using a Python package, DeepLabCut (Mathis et al., 2018; Nath et al., 2019), several marker-less body points of fish were automatically tracked. First, approximately 100 frames were extracted from videos of each camera to manually digitize the tip of the fish's snout, its eye, the centre of the body, and its tail to create a training dataset. With those datasets, a deep neural network was trained. Body points from all videos were tracked automatically using the trained model, producing *x–y* coordinates (pixel coordinates) in each frame and *P*-values that show the probability of the model prediction. Tracked points with *P*-values less than 0.6 and 0.9 for cameras above and beside the flume, respectively, were removed and interpolated with a cubic spline interpolation. The lower *P*-value was chosen for the camera above the flume because occlusion of body points rarely occurred and a *P*-value >0.6 still produced correct tracking results. Because DeepLabCut treats each frame independently, tracked points sometimes exhibit unrealistic jumps in time, which cannot be fully corrected using a *P*-value cutoff. To identify these outliers, we used the Hampel filter with a window size of 29 and a threshold corresponding to the median±s.d. Parameters of the Hampel filter were chosen by confirming that most unrealistic jumps were corrected. Detected points were removed and interpolated using cubic spline interpolation. Finally, data were smoothed with the running mean filter with a window size of five (0.056 s).

To reconstruct body points in 3D from 2D video data, cameras were first calibrated. Camera calibration consisted of intrinsic and extrinsic calibration. Intrinsic calibration concerned camera properties that were independent of camera position, such as the focal length, image size and location of the principal point of each camera. Intrinsic calibration for each camera was performed using the OpenCV package in Python with videos of a checkerboard moving toward the camera. Extrinsic properties involved positions and orientations of the two cameras and were estimated with a MATLAB package, easyWand5, which returns DLT coefficients (Theriault et al., 2014). To perform extrinsic calibration, easyWand5 requires videos with a wand swinging over the field of view, a picture of dotted grid paper, a picture of a box with markers specifying the origin and three axes and intrinsic properties of each camera. Using DLT coefficients obtained from extrinsic calibration, two-dimensional coordinates from videos were transformed into 3D coordinates (Hartley and Zisserman, 2004).

### Estimation of vectorial dynamic body acceleration (VeDBA)

Acceleration axes of an accelerometer are in an animal's frame of reference, whereas video-based acceleration axes are in the Earth's frame of reference. Thus, we used vectorial dynamic body acceleration (VeDBA, vectorial sum of DBA) rather than overall dynamic body acceleration (ODBA), which sums absolute DBA values in three axes.

To examine the relationship with oxygen consumption rates, VeDBA was obtained from videos. In this study, VeDBA of the eye was computed and used for further analysis because marker-less tracking was susceptible to errors for other body points, which are sometimes not clearly distinguishable. Using the eye position time series in 3D, we employed the second order forward finite difference method to obtain instantaneous acceleration in each axis. VeDBA was calculated as:
(2)

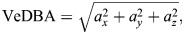
where *a_x_*, *a_y_* and *a_z_* are dynamic body acceleration values measured in three orthogonal axes in the Earth's frame of reference. Because acceleration measured by an accelerometer includes gravitational acceleration, DBA and gravitational acceleration are usually separated via moving average smoothing (Halsey et al., 2011). With the video-based method, however, raw acceleration corresponds to DBA because it does not include gravitational acceleration. The sampling rate was 90 Hz, which is the same as the frame rate of videos. In each measurement, mean VeDBA was computed. Even though data processing was applied to tracked body points, resulting 3D data still contained a few moments requiring adjustment. These moments included unrealistic jumps that remained after processing, as well as periods of consistently low VeDBA (<0.001 g), corresponding to intervals when tracked fish were outside the image frame. Thus, VeDBA values >1 g and <0.001 g were removed when averaging (1 g corresponds to the Earth's gravitational acceleration, 9.8067 m s^−2^). Mean VeDBA and oxygen consumption rates from the two replicates for each condition were used to test their relationship. In oxygen consumption experiments, we missed 3D data for 8 of 60 measurements because of camera malfunctions. In those cases, one of the two replicates for each condition was used as a representative.

### Oxygen consumption measurements during feeding

The relationship between oxygen consumption rates and VeDBA described above was obtained during steady swimming without feeding. To examine whether the same relationship holds during feeding, we further measured oxygen consumption rates with and without prey injection at three flow speeds (10, 20 and 30 cm s^−1^) for three individuals (A–C). Experiments were conducted on 17–20 October 2023.

Experiments were conducted following the aforementioned protocol except for differences in flow speeds and prey injection. In detail, each trial comprised a series of six measurement cycles at increasing flow speeds from 10 to 30 cm s^−1^ in increments of 10 cm s^−1^ and two measurements with and without prey injection at each flow speed. Each measurement cycle consisted of 5 min of flush, 2 min of equilibration and 20 min of measurement. During the second cycle at each flow speed, 1000 prey items were injected gradually over the 20 min measurement period. Background respiration measurements without fish were conducted both with and without prey injection to confirm a negligible impact of prey respiration. *Ṁ*_O_2__ and VeDBA were then computed as described.

### Statistical analysis

To test the relationship between oxygen consumption rates and VeDBA, we used a generalized linear mixed model (GLMM) fit with REML (restricted maximum likelihood), which accounts for random effects of dependent data (Bates et al., 2015). Data were analysed by specifying VeDBA as a fixed effect and individuals as a random intercept effect using the lme4 package (Bates et al., 2015) in R (r-project.org). Significance was computed with the lmerTest package (Kuznetsova et al., 2017), which performs analysis of variance to acquire *P*-values by applying the Kenward–Roger's degree of freedom method for mixed models. Marginal and conditional *R*^2^ values were further computed. To test whether inclusion of individuals as a random intercept effect produces a better model, a likelihood ratio test was conducted between models with and without the random effect. Because VeDBA is an activity-based proxy, relationship between net cost of swimming (*Ṁ*_O_2__–SMR) and VeDBA was tested in the same way.

## RESULTS AND DISCUSSION

### Video-based VeDBA as a proxy of oxygen consumption rates

**Fig. 1.**
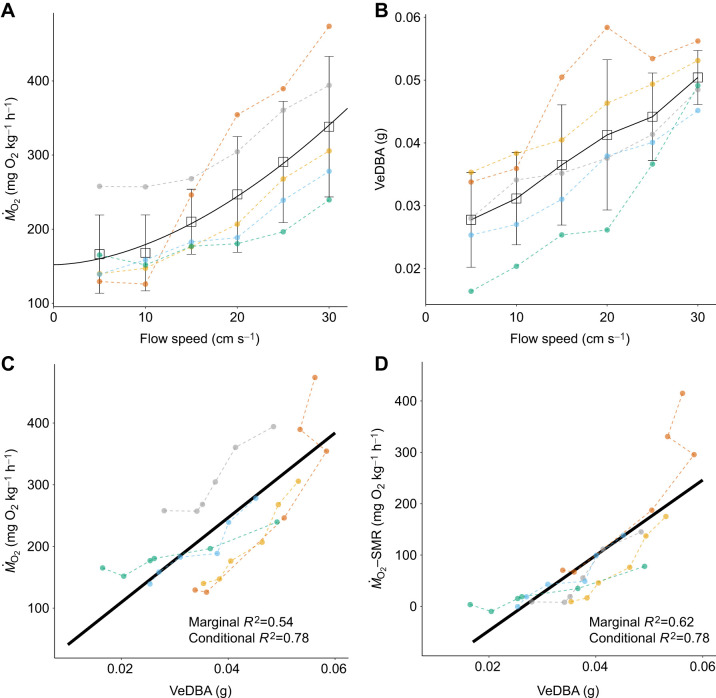
**Relationship between rates of oxygen consumption *Ṁ*_O_2__ and VeDBA.** *Ṁ*_O_2__ and VeDBA increased with flow speed. Colours indicate different individuals. Open squares and error bars are means±s.d. of five individuals. The solid line indicates (A) the fitted power function (*y*=*a*+*bx^c^*, *P*<0.001), (B) the connecting line, and (C,D) GLMM with VeDBA as a fixed effect and individuals as a random intercept effect (*P*<1×10^−6^).

Video-based VeDBA was examined for its relationship with oxygen consumption rates at a range of flow speeds. Both increased with increasing flow speeds (Fig. 1A,B; *Ṁ*_O_2__=152.15+0.47^1.76^). Based on the GLMM analysis, there was a significant relationship between oxygen consumption rates and VeDBA (Fig. 1C; *P*<1×10^−6^). Marginal *R*^2^ (only fixed term) was 0.54, and conditional *R*^2^ (fixed term+random term) was 0.78, corroborating the use of video-based VeDBA as a proxy for oxygen consumption rates. The likelihood ratio test showed an improved power of that proxy when individuals were included as a random factor, suggesting that the model's intercept depends significantly on individuals. Thus, when individual differences are considered, VeDBA becomes a better proxy, corresponding with previous findings (Allen et al., 2022; Green et al., 2009).

Because VeDBA is based on movement of an individual, its power as a proxy of metabolic costs decreases as the proportion of non-activity cost increases. In respirometry studies of fish, when oxygen consumption rate is plotted against flow speed and fitted with a power function, the oxygen consumption rate at zero flow speed is considered the standard metabolic rate (SMR) which is the minimum amount of energy required for maintenance, i.e. the resting metabolic rate (Bushnell et al., 1994; Reidy et al., 2000; Roche et al., 2013). Therefore, we examined the SMR by fitting the power function for each individual. We then computed the net cost of swimming by subtracting SMR from oxygen consumption rates, after which we examined its relationship with VeDBA. GLMM analysis indicates a significant relationship between net cost of swimming (*Ṁ*_O_2__–SMR) and VeDBA (Fig. 1D; *P*<1×10^−6^). Marginal *R*^2^ (the only fixed term) was 0.62, and conditional *R*^2^ (fixed term+random term) was 0.78, suggesting that VeDBA estimates activity-related energy cost better than total cost, which includes SMR.

**Fig. 2.**
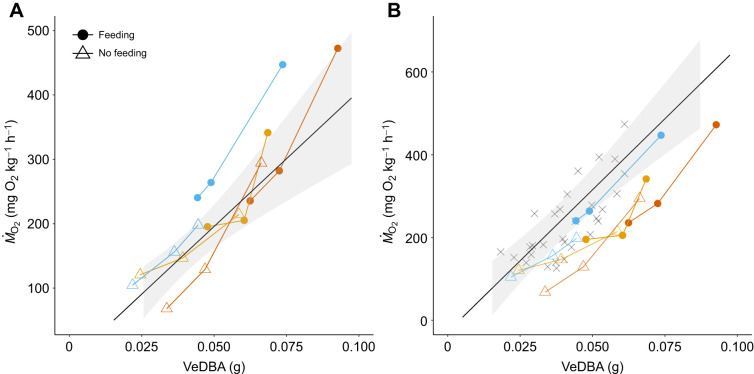
**Relationship between rates of oxygen consumption *Ṁ*_O_2__ and VeDBA during feeding can be estimated from models during steady swimming.** Colours represent individuals. Open triangles show data points during steady swimming and filled circles show data points during feeding. Shaded areas represent 95% confidence intervals. (A) Data points during feeding mostly overlapped with the GLMM during steady swimming (*P*<0.01). (B) Data points in A show a shift toward lower *Ṁ*_O_2__ at the same VeDBA compared with the GLMM from Fig. 1C (*P*<1×10^−6^). Cross symbols refer to data points in experiments shown in Fig. 1C.

To understand whether a model during steady swimming can be applied to other types of behaviour, we conducted a similar experiment, but with and without prey to induce feeding behaviour and steady swimming, respectively. During steady swimming, VeDBA and oxygen consumption rates were significantly related (open triangles and GLMM in Fig. 2A, *P*<0.01). During feeding, both VeDBA and oxygen consumption rates at each flow speed were higher than those during steady swimming, yet data points obtained during feeding mostly fell onto the model made from measurements during steady swimming (Fig. 2A). This result suggests that models made during steady swimming can be applied to estimate oxygen consumption rates during feeding.

Compared with the model in Fig. 1C, data points in this experiment showed a shift toward lower oxygen consumption rates at the same VeDBA (Fig. 2B). This shift may be caused by base conditions of fish affected by seasonal differences and temperature differences in a holding tank (Beamish, 1964). Thus, care should be taken when there are expected differences in animal conditions between calibration and application periods.

DBA is highly correlated with oxygen consumption of a wide range of animal taxa, including humans, mammals, birds, reptiles, amphibians, fish, scallops and lobsters (Halsey et al., 2009, 2011; Lyons et al., 2013; Payne et al., 2011; Robson et al., 2012; Wilson et al., 2020). Because of its simplicity in conducting measurements with high temporal resolution, the DBA method has been widely used in studies addressing metabolic costs of diverse behaviors in different ecological contexts (Grémillet et al., 2018; Rotics et al., 2016; Williams et al., 2014). Usually, relationships between DBA and oxygen consumption are first examined in laboratories to make calibration curves, which in turn, are used to estimate energy costs in field observations (Wilson et al., 2020). Because the conventional method to measure DBA requires attachment of a logger, estimation of metabolic costs through DBA has been limited to animals larger than a few hundred grammes. The video-based method we present in this study does not suffer from that limitation. Our application of this method to damselfish in the laboratory can be extended to the field as well as to many other small animals on land and in the aquatic environment. Also, because calibration curves are made in laboratories, which limit the range of movements, accuracy is sometimes compromised when applying the curve to different types of movement (Bidder et al., 2017). Here, we demonstrate that the model for steady swimming can be robustly used to estimate energy expenditure during feeding (Fig. 2A).

### Comparison with accelerometer-based VeDBA

DBA measured using an accelerometer and image processing are theoretically the same, which led us to develop the present video-based method. Acceleration values measured with an accelerometer include gravitational acceleration, independent of animal movement. DBA is derived by subtracting the running mean of gravitational acceleration from raw acceleration data (Shepard et al., 2008). Some cases include fast manoeuvres, in which computed gravitational acceleration is not equal to 1 g because of increased inertial acceleration (Wilson et al., 2013). Since the video-based method does not include gravitational acceleration, the recorded acceleration is only from animal movements. Errors of DBA measured by a logger are also caused by calibration, position, and stabilization level of the logger (Garde et al., 2022), which all come from attachment of a logger and are not a concern when using the video-based method.

The video-based method has some limitations. While the error range is usually consistent in an accelerometer, the error of the video-based method is case-specific, related to the configuration of cameras, tracking and 3D reconstruction. Therefore, the size of tracked body points in frames should not differ too much between calibration and application. Resolution, light conditions and camera settings may also affect tracking errors. Because DeepLabCut is used for automatic tracking, accuracy of the model also affects results. Post-processed results, in particular, should be compared with videos after automatic tracking to ensure their correspondence with actual movement and parameters of filtering should be adjusted as needed. Furthermore, calibration of the 3D reconstruction should be conducted properly to minimize errors. To minimize such errors, we suggest that calibration curves should be constructed in the laboratory under conditions that are as close as possible to those applied. However, the system-dependent error range becomes advantageous when the method is applied to microscale animals, as it allows users to determine the subject's size in an image, even under a microscope, and it enables precise measurement of acceleration.

The basic assumption underlying use of DBA is that a major part of total energy cost depends on animal movements. Thus, as with the accelerometer-based method, our video-based method cannot estimate non-movement-based energy cost, such as thermoregulation, stress and isometric muscle contraction. Care should be taken to interpret data when large effects of these energy costs are expected.

### Applications

DBA has been used mainly to estimate field metabolic rate (Grémillet et al., 2018; Rotics et al., 2016; Williams et al., 2014; Wilson et al., 2006). Similarly, a video-based method may allow estimation of oxygen consumption rates in the field when animals stay mostly in the field of view. For instance, our subject species, *C. viridis*, always remains close to its home coral colony, for shelter from predators. If arranged appropriately, cameras are able to capture their behaviour full-time and acceleration of different behaviours can be measured. However, animals in the field often move far away or stay in places outside the field of view, preventing recordings of their complete movements. Thus, our method is robust in laboratory experiments where users can control these unwanted situations. Also, as suggested by our results, calibration for each individual should be performed and used in an application to achieve higher accuracy, which can easily be done in laboratory experiments. Although respirometry in a flume or chamber has been a standard method to estimate fish energy cost in the laboratory, it cannot measure energy cost of some behaviours because of constraints imposed by the chamber. For example, individual energy cost during fish schooling behaviour has been of interest in behavioural ecology research. However, it cannot be measured by respirometry because it is not possible to identify the energy cost of each individual and also because of a conflict between the small water volume in a respirometer and the large working volume to minimize wall effects on fish schooling (Johansen et al., 2010; Noda et al., 2016). Moreover, natural feeding requires volumes larger than are available in respirometers (Kiflawi and Genin, 1997) and it is expected to require much more energy than steady swimming as it involves rapid acceleration and deceleration by turning and striking. Fin beat frequency was used as a proxy of energy expenditure (Johansen et al., 2010; Tudorache et al., 2009), but such proxies during steady swimming in a respirometer may under-represent the other behaviours that involve rapid changes in speed and trajectory (Gleiss et al., 2010; Marcoux and Korsmeyer, 2019; Tang et al., 2000; Trudel and Boisclair, 1996). With the video-based DBA method, energy costs of these behaviours can be estimated on an individual basis without the volume limitation.

By combining recent advances in automatic tracking techniques and dynamic body acceleration, we developed a video-based method to estimate energy expenditure of animals. This approach enables us to estimate energy costs without limitations, such as confinement in small chambers and attachment or implantation of loggers. This technique may be applied to any animals, but is especially useful for small animals, for which the conventional DBA method with an accelerometer cannot be used.
